# Parasite Glycobiology: A Bittersweet Symphony

**DOI:** 10.1371/journal.ppat.1005169

**Published:** 2015-11-12

**Authors:** Joao A. Rodrigues, Alvaro Acosta-Serrano, Markus Aebi, Michael A. J. Ferguson, Françoise H. Routier, Irene Schiller, Simão Soares, Daniel Spencer, Alexander Titz, Iain B. H. Wilson, Luis Izquierdo

**Affiliations:** 1 Instituto de Medicina Molecular, Faculdade de Medicina da Universidade de Lisboa, Lisboa, Portugal; 2 Department of Parasitology & Department of Vector Biology, Liverpool School of Tropical Medicine, Liverpool, United Kingdom; 3 Institute of Microbiology, Department of Biology, Swiss Federal Institute of Technology, ETH Zurich, Zurich, Switzerland; 4 Division of Biological Chemistry and Drug Discovery, The College of Life Sciences, University of Dundee, Dundee, United Kingdom; 5 Hannover Medical School, Hannover, Germany; 6 Malcisbo AG, Zurich, Switzerland; 7 SilicoLife Lda., Guimarães, Portugal; 8 Ludger Ltd., Culham Science Centre, Abingdon, Oxfordshire, United Kingdom; 9 Helmholtz Institute for Pharmaceutical Research Saarland (HIPS), Saarbrücken, Germany; 10 Universitaet fuer Bodenkultur Wien, Austria; 11 ISGlobal, Barcelona Centre for International Health Research (CRESIB), Hospital Clínic - Universitat de Barcelona, Barcelona, Spain; University of Wisconsin Medical School, UNITED STATES

Human infections caused by parasitic protozoans and helminths are among the world's leading causes of death. More than a million people die each year from diseases like malaria and neglected tropical diseases like leishmaniasis, trypanosomiasis, and schistosomiasis. Patients also endure disabilities that cause lifelong suffering and that affect productivity and development [1]. More insidiously, parasites generate important economic losses, since they often also infect commercially valuable animals. Worldwide, exposure to parasites is increasing due to growing international travel and migrations, as well as climate changes, which affect the geographic distribution of the parasite vectors. The parasitic threat is also aggravated by the rise of the immunocompromised population, which is particularly sensitive to parasite infections (e.g., individuals with AIDS and other immunodeficiencies).

A common feature of protozoan parasites and helminths is the synthesis of glycoconjugates and glycan-binding proteins for protection and to interact and respond to changes in their environment. To address the many challenges associated with the study of the structure, the biosynthesis, and the biology of parasitic glycans, the authors of this article have established GlycoPar, a European Marie Curie training program steered by some of the world's academic leaders in the field of parasite glycobiology, in close association with European industrial enterprises. The main scientific goal of this network is the description of novel paradigms and models by which parasite glycoconjugates play a role in the successful colonization of the different hosts. By means of a training-through-research program, the aim of the network is to contribute to the training of a generation of young scientists capable of tackling the challenges posed by parasite glycobiology.

## Parasites Are Covered by a Protective Glycocalyx

**Fig 1:**
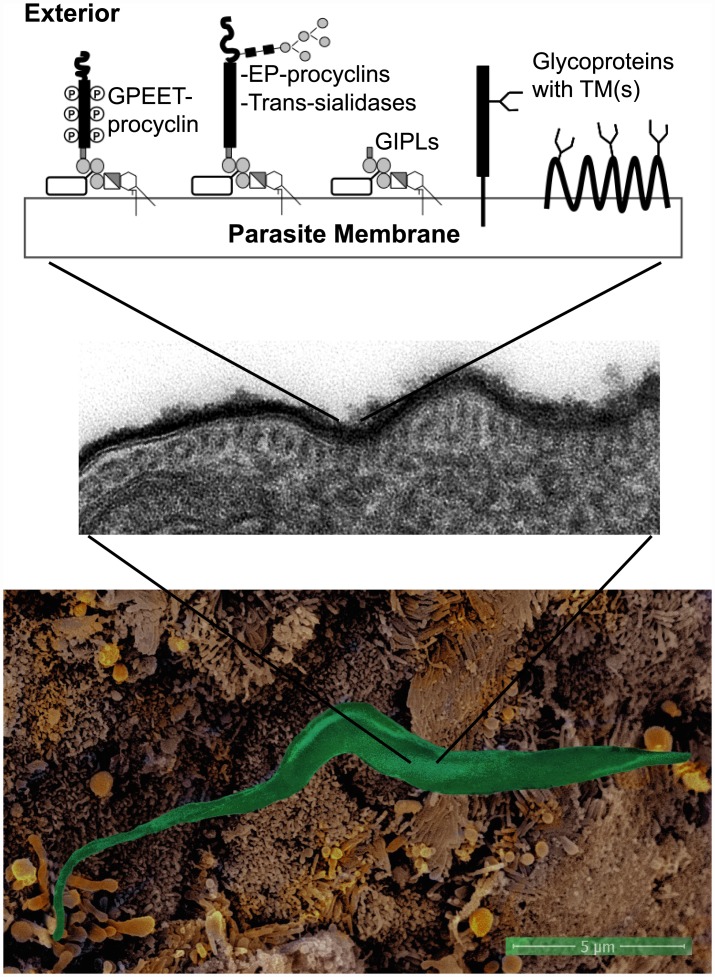
The surfaces of parasites, such as *Trypanosoma brucei brucei*, are covered by glycoconjugates forming a protective glycocalyx against the host defense systems. False-color scanning electron microscopy (EM) of a *T*. *b*. *brucei* procyclic interacting with cell microvilli in the tsetse fly proventriculus (bottom panel). Transmission EM of ruthenium-red stained ultrathin sections showing the surface glycocalyx of *T*. *b*. *brucei* procyclic cells (middle panel). Scheme summarizing the main surface glycosylphosphatidylinositol (GPI)-anchored (EP- and GPEET-procyclins and trans-sialidases) and transmembrane (including polytopic) glycoproteins and glycolipids expressed by *T*. *b*. *brucei* procyclics (top panel) [2,24]. Open rectangles linked to GPI molecules represent side chains characteristic of surface glycoconjugates from procyclic *T*. *b*. *brucei*. GIPLs: glycoinositolphospholipids, or free GPIs. EM images obtained by C. Rose, A. Beckett, L. Tetley, I. Prior, and A. Acosta-Serrano.

Due to the complexity of their life cycles, parasites need to sequentially exploit various host species to complete the different stages involved in their survival and development. The interactions with their different hosts are critical for the completion of each life stage and are often based on carbohydrate recognition. In particular, parasites have developed different strategies to escape the immune and defense systems of the different infected organisms. Their surfaces are covered by glycoconjugates of varied natures, often of types absent from mammals. This so-called glycocalyx is protective against the host defense systems but may also be implicated in "hijacking" proteins involved in host innate immunity (Fig 1) [2,3]. Thus, through a "glycan gimmickry" designated process, helminths express host-like glycans that interact with host lectins to modulate the immune response [4]. Furthermore, the walls that protect different parasitic cysts from harsh environments are also rich in polysaccharides and polysaccharide-binding lectins [5]. Thereby, glycans are crucial for parasite virulence and survival.

Since glycans are central to host–parasite interactions, their study constitutes a fertile, but currently largely unexploited, area for therapeutic applications. Research in parasite glycosylation provides new opportunities for the discovery of vaccine candidates and for the development of novel chemotherapy approaches and diagnostic tools. Thus, for instance, besides its effect modulating the host immune response against the infection, glycans from *Schistosoma mansoni* are currently being explored as targets for vaccination and/or serodiagnosis of human schistosomiasis [6]. Nevertheless, there are many challenges associated with working with parasites, including problems in obtaining sufficient amounts of biological material for analytical purposes, difficulties of culturing the different life stages, and, on occasion, the lack of tools for functional genomics and molecular biology approaches. Glycans add another level of difficulty to these studies, due to their extensive diversity and exquisite complexity. In contrast to nucleic acid and proteins, their biosynthesis is only indirectly template-driven and generates an important amount of structural variability in biological systems. This complexity is critical in molecular recognition events including cell–cell, cell–matrix, and cell–molecule interactions during essential steps of pathogenesis. Thus, the thorough characterization of parasite glycobiology requires systematic approaches that focus on the description of the glycosylation precursors, the glycan-processing enzymes, and the structure and functional significance of parasitic glycans. In addition, most of the medically and veterinarially important parasites are phylogenetically ancient organisms and represent good models for studying evolutionary aspects of eukaryotic glycosylation. Thus, the study of parasite glycans may unravel novel mechanisms also present in higher eukaryotes. Excellent examples are the description of the structure of glycosylphosphatidylinositol (GPI) membrane anchors in African trypanosomes [7] or the discovery of the glycoprotein quality control cycle, thanks to seminal studies on the N-glycosylation pathway of trypanosomatid parasites [8]. Interestingly enough, different parasitic protists present variable lengths in their N-glycan precursors that directly affect this N-glycan-dependent quality control system [9].

## The Metabolic Precursors of Parasite Glycosylation

Glycan synthesis requires activated monosaccharides, mainly in the form of nucleotide sugars that will be used by glycosyltransferase enzymes as glycosyl donor substrates in glycosylation reactions. Therefore, the presence of activated sugars is a prerequisite for glycan biosynthesis, and their availability influences the glycan structures that may be synthesized by a parasite (the glycome). Thus, valuable information about the glycome can be gained from the identification and quantification of the sugar nucleotide pools maintained during the life stages of different parasites. For example, the capping of *Leishmania major* surface lipophosphoglycan with arabinose side chains, which is required for detachment of the infectious parasites from the sand fly midgut, correlates with a strong increase of the GDP-α-D-arabinopyranose pool [10].

**Fig 2:**
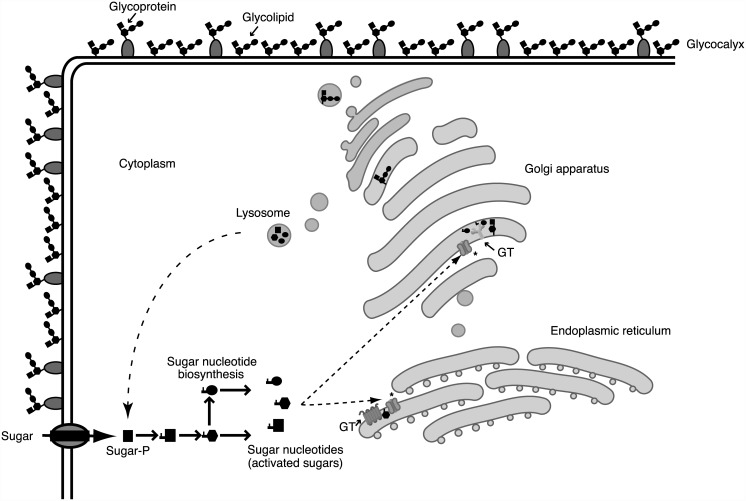
Glycosylation processes involve different cellular compartments. Glycan biosynthesis and cellular compartments involved in the glycosylation process. Sugars are carried across the plasma membrane into cells or are salvaged from degraded glycoconjugates at lysosomes. Through biosynthetic and interconversion reactions, monosaccharides are activated into different nucleotide sugars. Sugar activation generally takes place in the cytoplasm, although several enzymes involved in sugar nucleotide biosynthesis in *T*. *b*. *brucei* are localized in the glycosome. After being activated, sugar nucleotides are transported into the endoplasmic reticulum/Golgi apparatus and used by different glycosyltransferases (GT). Glycosyltransferases and other glycan-processing enzymes define the assembly and final structure of glycans that are secreted or located in the cell surface, forming a protective glycocalyx. Sugar nucleotide transporters are marked with an asterisk (*).

Sugar nucleotides are formed by de novo pathways requiring the bioconversion of an existing sugar or sugar nucleotide or by salvage pathways involving the activation of the sugar using a kinase and a pyrophosphorylase. The conservation of specific biosynthetic pathways in the parasite genomes are strong hints of the presence of nucleotide sugar pools [11,12]. Monosaccharide activation usually takes place in the cytoplasm, although in *Trypanosoma brucei brucei* and possibly other kinetoplastid parasites, these biosynthetic reactions occur in a specific organelle called glycosome [13]. Since sugar nucleotides are mostly used by glycosyltransferases in the endoplasmic reticulum and/or the Golgi apparatus, they must be translocated to these cellular compartments by specific transporters (Fig 2). This metabolic compartmentalization and the study of the transporters involved also offer new opportunities for the selective inhibition of crucial glycosylation reactions in parasites.

## Parasitic Glycan-Processing Enzymes and Glycan-Binding Proteins

Glycosyltransferases transfer sugar moieties from activated donors to specific acceptor molecules, generating glycosidic linkages between carbohydrates or between a carbohydrate and a noncarbohydrate moiety. Therefore, they define the assembly and final structure of glycan chains, which can be linear or branched and of various lengths. Glycoside hydrolases, the enzymes that hydrolyze glycosidic bonds, form another main group of carbohydrate-active enzymes that also play important roles in determining the final structure of mature glycans. The combined action of several of these enzymes in the secretory pathway leads to a vast and diverse array of glycan structures. Additionally, parasitic glycan-binding proteins interact with specific parasite and host glycan structures present in the surface of cells.

Sequence-based families of glycosyltransferases, glycoside hydrolases, and carbohydrate-binding proteins group together according to their function, indicating that the acquisition of the specificities of these enzymes evolved from common progenitors. Therefore, despite the huge diversity of glycans, the activities and molecular mechanism of the enzymes involved in their biosynthesis can often be inferred from their sequences [14]. Nevertheless, because of the substantial evolutionary distance between protozoan parasites and higher eukaryotes, it can be challenging to define the precise function of specific parasitic glycosyltransferases from sequence similarity [15,16] or by inference from the final structures determined by a particular glycosylation pathway [17,18]. Glycosyltransferases and other glycan-processing enzymes involved in the biosynthesis of glycans essential for the survival and infectivity of parasites might be exploited as drug targets. Therefore, increasing our knowledge of the different parasitic glycosylation pathways and their biological relevance will contribute to uncovering the therapeutic potential therein.

## Parasite Glycomics and the Biological Function of Glycoconjugates

The characterization and quantification of the complete set of glycans and glycoconjugates made by a cell or organism at a given time is defined as glycomics. Since glycosylation is the most structurally diverse, and one of the most abundant, protein and lipid modifications, the description of the spectrum of all glycan structures—the glycome—of even just a single cell type is a huge challenge. Nevertheless, to shed light on the structure–function relationship of parasite glycans at the molecular level, a detailed knowledge of their structures is an important prerequisite that can only be achieved through the use of different analytical methodologies and glycoproteomics and glycolipidomics strategies. Currently, mass spectrometry is a key tool in glycomics and has revealed highly unusual glycans from a number of unicellular and metazoan parasites [19].

The assessment of the functional significance of the different glycosylation states will only be achieved by employing adequate screening and/or genetic tools that, in the case of particular parasites, are still in the development stage [20]. Host receptor molecules can specifically recognize glycans, and these glycan–receptor interactions are related to migration, invasion, adhesion, toxin production, and other essential processes during the course of parasitic infections. By a thorough exploration of the glycomic capacity of parasites and its influence on the interactions with their hosts, the code defined by the different glycan structures can be gradually characterized. In addition, glycomic approaches can be illuminating in the discovery of novel antigenic glycans for the development of diagnostic tools or glycovaccines. An important step in this respect would be the development of glycan microarrays reflecting parasite glycomes in order to identify binding partners in the human proteome, such as components of the innate immune system. Similarly, identifying host glycan structures recognized by parasite proteins with lectin-like properties will be fundamental for describing host–parasite interactions in parasitic diseases.

## Future Perspectives: The Translation of Parasitic Glycobiology

Glycobiology has become a well-established area of study in recent decades and is currently providing drug targets against several pathogens and diseases. Ethambutol, Caspofungin, Zanamivir, and Oseltamivir are well-known examples of commercial drugs in use—as therapies against tuberculosis, candidiasis, aspergillosis, and influenza—that target glycosylation and carbohydrate processing. In this regard, echinocandins, antifungal drugs that target β-1,3-glucan synthesis, also inhibit oocyst wall biosynthesis in *Eimeria* [21]. Similarly, bacterial polysaccharide–protein conjugate vaccines have recently revolutionized vaccination strategies. This approach may be applied to prevent or treat parasitic diseases, using parasite-derived xeno-glycans absent in the human glycome [6,22]. Furthermore, the identification of parasitic glycan antigen structures and monoclonal antibodies to these epitopes holds unprecedented promise for the development of novel diagnostic procedures for various parasitic infections [23]. Thus, through profound and systematic approaches to this important but frequently neglected area of pathogenic parasite research, knowledge about the biology of these organisms will be extended, and novel methods to tackle them will likely be uncovered.
